# Spousal Support for Patients With Rheumatoid Arthritis: Getting the Wrong Kind Is a Pain

**DOI:** 10.3389/fpsyg.2018.01760

**Published:** 2018-09-20

**Authors:** Jessie Pow, Ellen Stephenson, Mariët Hagedoorn, Anita DeLongis

**Affiliations:** ^1^Department of Psychology, Centre for Health and Coping Studies, The University of British Columbia, Vancouver, BC, Canada; ^2^Department of Health Psychology, University Medical Center Groningen, University of Groningen, Groningen, Netherlands

**Keywords:** social support, dyadic coping, pain, rheumatoid arthritis, solicitous support, emotional support, negative support, intensive longitudinal methods

## Abstract

Research indicates that perceived support availability is beneficial, with support available from the spouse particularly important for well-being. However, actual support mobilization has shown mixed associations with recipient well-being. The primary goal of the present study was to go beyond examining the effects of global perceptions of support on recipient outcomes. Instead, we examined the effects of several specific types of support that have been found to be important in the clinical literature. In this study, we followed both members of couples in which one partner was diagnosed with rheumatoid arthritis. Patients provided reports on pain for both mornings and evenings across 1 week. Both partners also reported esteem, solicitous, and negative support mobilization received by the patient. We found that patient pain tended to increase across the day following increases in patient reports of negative support receipt and partner reports of solicitous support provision. We also found that patient pain tended to decrease across the day when partners reported increased levels of esteem support provision. Reverse causation analyses indicated higher levels of patient pain may lead partners to increase solicitous support mobilization to the patient. Findings underscore the importance of examining both partners’ reports of support within a dyadic coping framework. They further suggest that not all forms of support are equally beneficial, calling for a finer grained assessment of specific support transactions.

## Introduction

What is the best way for the spouse to provide support? Should he express how concerned he is about the patient? Should she assure her spouse that he is loved, valued, and important? Do his avoidant or critical responses influence his partner’s well-being over time? Dyadic coping theory suggests that spouse responses play a critical role in influencing well-being (Bodenmann et al., 2007), especially for those coping with chronic illness (Revenson and DeLongis, 2010). However, the literature is mixed regarding the effectiveness of support. Some studies find beneficial effects of support receipt (Pasch and Bradbury, 1998; DeLongis et al., 2004), whereas others find no effects (Barrera, 1986; Bolger et al., 1996) or even detrimental effects of support receipt (Bolger et al., 2000; Jang et al., 2003; Shrout et al., 2006; Gleason et al., 2008). Differences in findings may be attributable to limitations due to aggregating across multiple types and instances of support as well as limitations inherent in only examining one partner’s perspective. In this paper, we addressed these issues by using an intensive longitudinal design to examine reports of several types of partner support mobilization in predicting subsequent pain among patients with rheumatoid arthritis (RA). We took a dyadic perspective by examining the perceptions of both RA patients and their partners.

Although research has emerged accounting for the role of both partners’ perceptions of support in key psychosocial outcomes (Bodenmann et al., 2006; Badr and Taylor, 2009; Rosen et al., 2014, 2015), the impact of relationships on chronic illness has most often been investigated via patient reports only (Revenson and DeLongis, 2010). In couples in which one partner is coping with a chronic illness, the impact of both partners’ responses on patient outcomes is not well understood. However, previous studies of RA patients and their spouses underscore the importance of examining the role of spouse variables in patient disease course. For example, in a prospective study of RA patients and their spouses, spouse reports of their own depressive symptoms predicted increased functional limitations and RA-related symptoms for patients over a one-year period, controlling for earlier patient depression and functional limitations or RA-related symptoms (Lam et al., 2009; Stephenson et al., 2014).

Outside of chronic pain contexts, studies suggest the support recipients’ and support providers’ perceptions both independently predict changes in recipient well-being. For example, in one study examining global levels of support, provider reports of emotional support were associated with decreases in negative affect, but recipient reports of emotional support were associated with increases in negative affect (Bolger et al., 2000). Other studies suggest that recipients and providers have unique perspectives that may jointly provide important insights into the role of support (Collins and Feeney, 2000).

Many studies on support transactions in intimate relationships have examined support as a global construct (Bolger et al., 2000; DeLongis et al., 2004). However, theoretical models outline several types of support, which are expected to have different implications for well-being (Schulz and Schwarzer, 2000; Cano et al., 2008). The focus on global levels of support mobilization is a limitation of the literature because it leads to an incomplete understanding of which specific transactions have occurred. This limited focus does not offer insights into which specific behaviors to target with interventions promoting adaptive responses to stress in couples. In the current study, we examined three types of support. First, we examined *esteem/emotional support*, which refers to expressions that the recipient is loved, valued, and accepted. The second type of support we examined was *solicitous support*, which involves conveying concern for the support recipient (Flor et al., 1989; Newton-John, 2002). Third, we examined *negative support*, which includes being critical of the support recipient or avoiding the support recipient (Bodenmann, 2005; Sullivan et al., 2010). Studies indicate that these forms of support are distinct constructs (Cano et al., 2008; Brock et al., 2014) and that they may be key for couples coping with chronic pain (Hemphill et al., 2016).

There are two leading models that make different predictions regarding which types of support should be effective ways of promoting well-being for those experiencing chronic pain (Cano and Williams, 2010; Hemphill et al., 2016). Pain research has traditionally relied on operant models, which indicate that pain behaviors communicate pain to others, and others’ supportive responses to pain behaviors may inadvertently reinforce those behaviors (Fordyce, 1976), leading to an increase in pain. This model predicts that spousal emotional and solicitous support could reinforce pain behavior and lead to worse outcomes over time. This model also predicts that negative spouse responses extinguish pain behaviors and lead to better outcomes (Turk et al., 1992). In contrast to operant models, the interpersonal model predicts that spouse responses aimed at understanding and validating the patient’s emotions and pain experiences are intimacy-building, help individuals regulate emotions, and lead to better outcomes over time (Holtzman and DeLongis, 2007; Cano et al., 2008; Cano and Williams, 2010; Hemphill et al., 2016). Interpersonal models classify negative spouse responses as unsupportive and suggest that they undermine intimacy, disrupt emotion regulation, and lead to poorer outcomes (McCracken, 2005; Cano et al., 2008; Cano and Williams, 2010; Hemphill et al., 2016).

There is partial empirical support for both the traditional operant model and the interpersonal model of pain. Findings regarding solicitous support tend to be consistent with a traditional operant model. Several cross-sectional studies of couples coping with chronic pain indicate that individuals who receive higher levels of solicitous support from their partners tend to have worse well-being than those who receive lower levels of solicitous support (Romano et al., 1995, 2000; Fillingim et al., 2003; Boothby et al., 2004; McCracken, 2005). In contrast, findings for negative and esteem support tend to support the interpersonal model. Negative responses have been linked to poorer patient outcomes, including greater emotional distress, pain, and pain catastrophizing, as well as less activity engagement and lower acceptance of pain (Kerns et al., 1990; Keefe et al., 2003; Boothby et al., 2004; Cano, 2004; McCracken, 2005). Fewer studies have examined associations between esteem/emotional support and well-being in individuals with chronic illness. However, in one study, participants with osteoarthritis who perceived higher emotional support availability tended to report poorer functional ability (Weinberger et al., 1990).

Despite several cross-sectional studies examining associations between spouse responses and patient well-being, few studies have examined within-couple or prospective associations. In addition, there has been very little research examining spouse reports. These are key limitations of the literature because this makes it difficult to know whether partners change the amount and type of support they provide in response to worsening patient symptoms. For example, is it possible that solicitous support is linked to worse patient well-being because spouses increase their provision of solicitous support when their partners are experiencing more symptoms?

In a recent study of patients with osteoarthritis, Hemphill et al. (2016) examined change in physical limitations and physical activity over 6 and 12 months as a function of spouse reports of emotional support provision, solicitous support provision, and negative support provision to patients. Emotional support provision as reported by spouses was a significant predictor of subsequent decreases in functional limitations and increases in physical activity over 6 months; solicitous responses were significantly associated with increases in functional limitations and decreases in physical activity over 12 months. Negative support was not significantly associated with changes in patient outcomes over time. Although this study provides evidence for a beneficial effect of emotional support and a detrimental effect of solicitous support over time, the study did not examine reports from both partners, nor did the authors examine pain as an outcome. Given this, questions remain about whether spouse reports provide complementary information beyond patient reports, and whether each type of support similarly influences different outcomes.

Intensive longitudinal studies allow for within-couple examination of time-ordered associations among spouse responses and patient well-being. There have been only a few intensive longitudinal studies examining daily associations between patient well-being and esteem/emotional, solicitous, and negative partner responses. These studies tend to find benefits of emotional support and detrimental effects of solicitous and negative support on patient well-being (Badr et al., 2013; Rosen et al., 2014; Song et al., 2015). For example, in an intensive longitudinal study examining spouse responses to vulvodynia pain, patients’ sexual functioning improved on days when they reported receiving higher levels of emotional, lower solicitous, and lower negative support from their partners (Rosen et al., 2014).

A handful of studies have used intensive longitudinal methods to examine associations between spouse responses and patient pain (Holtzman and DeLongis, 2007; Burns et al., 2013; Rosen et al., 2015). In one study focusing on couples in which one partner had chronic low back pain, Burns et al. (2013), found that patient perceptions of higher spouse hostility and criticism were both associated with higher concurrent patient pain when controlling for prior pain intensity. Additionally, patient perceptions of higher spouse hostility were associated with residualized increases in patient pain over the subsequent 3 h. However, no association was found between patient perceptions of spouse criticism and subsequent changes in patient pain. Although this study provides some evidence that negative spouse responses are prospectively associated with changes in patient pain, emotional, and solicitous spouse responses were not examined. Additionally, only patient reports of spouse responses were examined.

One intensive longitudinal study examined associations between both partners’ reports of spouse emotional, solicitous, and negative support and patients’ reports of vulvodynia pain. In this study, pain decreased on days when patients reported receiving lower levels of solicitous and negative support (Rosen et al., 2015). Patients’ pain decreased on days when their partners reported providing higher levels of esteem/emotional and lower levels of solicitous support (Rosen et al., 2015). Although this study provides initial evidence for beneficial effects of esteem/emotional and detrimental effects of solicitous and negative support on daily patient pain, prospective associations were not examined. Therefore, a viable alternative explanation of the findings is that increases in patient pain lead partners to change how they respond.

In this study, we examined the roles of both partners’ perceptions of esteem/emotional, solicitous, and negative support in predicting subsequent shifts in pain. We focused on couples in which one partner had been diagnosed with RA. RA is an incurable autoimmune disease that affects up to 1% of the global population (Woolf and Pfleger, 2003). It is associated with a number of debilitating symptoms, including chronic pain, stiffness and inflammation of the joints, fatigue, and frequent shifts in mood (Smith and Wallston, 1992). Given this, the spouse can play a key role in providing support to the affected individual.

We used an intensive longitudinal design (Bolger and Laurenceau, 2013) in which patients and their partners were asked to provide reports about 6 and 12 h after waking to examine the influence of specific types of spousal support mobilized in the morning on subsequent changes in pain from morning to evening. We predicted that mornings when higher levels of esteem support were mobilized than typical for that couple would be associated with subsequent decreases in pain. We also predicted that times when higher levels of solicitous and negative support were mobilized than typical for that couple would be associated with subsequent increases in pain. We expected that these associations would be maintained when morning levels of potential confounding variables were controlled, including the amount of time spent with the partner and mood.

We were also interested in examining, on an exploratory basis, whether spouses change their supportive behaviors in response to within-patient fluctuations in pain. Thus, we conducted reverse-causation analyses. In these analyses, we examined whether mornings when patients experienced higher levels of pain than typical for them were associated with subsequent shifts in esteem/emotional, solicitous, and negative support from the spouse.

## Materials and Methods

### Participants

Couples were recruited as part of a larger study on community-dwelling patients with RA (Holtzman and DeLongis, 2007; Beggs et al., 2015). This study is the first to report findings from the spouses of these participants. Eight-hundred potential study participants were randomly selected from a database of patients registered with the Mary Pack Arthritis Society and mailed an initial contact letter describing the study and inviting participation. The Mary Pack Arthritis Society is a local organization that offers treatment and education to arthritis patients across British Columbia, Canada. One hundred eighty-eight individuals contacted our research office and 160 agreed to be screened by telephone to ensure that they had been diagnosed with RA, experienced pain due to RA in the past month, and were able to read, write, and speak English. Participants in the current sample were also required to be living with a spouse or common law partner. Spouses were invited to participate following expressed interest by the patient. Of the 160 patients who agreed to participate in additional eligibility screening, 20 (13%) declined to participate, 17 (11%) were excluded because they had not experienced RA pain in the past month, and 52 (33%) were excluded because they were not married or living with a common law partner. Thus, 71 (44%) met inclusion criteria for the larger study of patients. Forty-one (26%) participated in the study but their spouse did not also participate and 30 patients (19%) both met inclusion criteria and had a spouse willing to participate. One of these couples had to be dropped from analyses because the patient never saw her spouse in the morning and therefore did not report morning support mobilization. Those who contacted our research office regarding their potential participation were entered into a draw for $1000. Additionally, all of those who met criteria and participated in the data collection phase were mailed a small gift valued at $10 CAD.

The final sample consisted of 29 couples (29 RA patients and 29 cohabitating spouses). Patients were mostly female (*n* = 21, 72%), which is consistent with sex differences in RA prevalence rates (Public Health Agency of Canada [PHAC], 2010), and Caucasian (*n* = 26, 90%), had children (*n* = 26, 90%), and had a mean age of 61.1 years (*SD* = 10.5, range = 42–82). The mean number of years since RA diagnosis was 17.7 (*SD* = 13.4, range = 1–50). Of the participating RA patients, seven (24%) were employed, twelve (41%) were retired, five (17%) were on sick leave, two (7%) were on disability, and three (11%) were homemakers at the time of the study. Patients and spouses had a relationship length averaging 31 years (*SD* = 15.8, range = 6 months – 59 years). Spouses of RA patients were mostly male (*n* = 19, 66%) and Caucasian (*n* = 25, 86%), with a mean age of 62.9 years (*SD* = 9.1, range = 46–85). Of the participating spouses, eleven (38%) were employed, thirteen (45%) were retired, one (3.7%) was on sick leave, and one (3.7%) was a homemaker at the time of the study. The modal family income was between $25,000 and $50,000 CAD.

### Procedure

Participants provided informed consent over the phone and then completed brief structured telephone interviews twice a day for 1 week, which were scheduled at approximately 6 and 12 h after waking up. At each interview, patients were asked to report on pain and support receipt from the spouse; spouses were asked to report support provision to the patient. These reports were in reference either to their experiences so far that day (for the morning assessment) or since the last interview (for the evening assessment). The twice daily phone interviews lasted approximately 10 min per interview and were administered by a trained female research assistant. Consistent interviewers were assigned to each participant to develop and maintain rapport. Participants were asked to find a private and quiet place in which to complete the daily interviews, and interviews were conducted separately with each member of the couple. With the permission of participants, all interview sessions were tape recorded and transcribed. Telephone methods were used (as opposed to electronic methods) due to the difficulties that may have arisen with holding and operating handheld devices and/or typing, given the previously noted functional disabilities and limitations common to individuals with RA. This study was approved by the affiliated institution’s Behavioral Research Ethics Board.

### Measures

#### Patient Pain

Patients reported intensity of pain associated with RA during the previous half-day using a numerical rating scale (NRS) from 0 (no pain) to 10 (pain as bad as it could be). The NRS has demonstrated positive and significant associations with other measures of pain intensity (Jensen et al., 1986; Wilkie et al., 1990) and sensitivity to treatments aimed at influencing pain intensity (Paice and Cohen, 1997).

#### Spouse Support Mobilization

Patients and their spouses provided reports on support mobilized from the spouse to the patient using a modified version of the Berlin Social Support Scales (BSSS; Schulz and Schwarzer, 2000). Participants provided responses on a 5-point Likert scale (0 = does not apply, 1 = not at all, 2 = a little, 3 = somewhat, 4 = a lot). We collapsed across the “does not apply” and “not at all” categories such that either response received a score of one. Patient esteem/emotional support receipt was assessed with two items (“He/she showed you that he/she loves and accepts you,” “He/she made you feel valued and important”; am *R*_c_ = 0.46; pm *R*_c_ = 0.56; all timepoints *R*_c_ = 0.53).^1^ Spouse esteem/emotional support provision was assessed with two parallel items (“You showed him/her that you love and accept him/her,” “You made him/her feel valued and important”; am *R*_c_ = 0.54; pm *R*_c_ = 0.48, all timepoints *R*_c_ = 0.55). Solicitous support receipt and provision were each assessed with two items (Patient receipt: “He/she comforted you when you were feeling bad,” “He/she expressed concern about your condition”; am *R*_c_ = 0.34; pm *R*_c_ = 0.53; all timepoints *R*_c_ = 0.47; Spouse provision: “You comforted him/her when he/she was feeling bad,” “You expressed concern about his/her condition”; am *R*_c_ = 0.54; pm *R*_c_ = 0.38, all timepoints = 0.45). Negative support receipt and provision were each assessed with two items (Patient receipt: “He/avoided you,” “He/she complained about you”; am *R*_c_ = 0.56; pm *R*_c_ = 0.25, all timepoints *R*_c_ = 0.48; Spouse provision: “You avoided him/her,” “You complained about him/her”; am *R*_c_ = 0.00; pm *R*_c_ = 0.00, all time points *R*_c_ = 0.00).^2^ If participants indicated that they had not seen or spoken to their spouse since the last diary entry, spouse support questions were skipped for that timepoint and treated as missing.

#### Control Variables

We asked patients to report the extent to which they saw or spoke to their partner (1 = Not at all, 4 = A lot). Positive and negative affect were assessed using the Affects Balance Scale (Derogatis, 1975). Positive affect was assessed with five items (am *R*_c_ = 0.82; pm *R*_c_ = 0.78; all timepoints *R*_c_ = 0.80). Negative affect was the combined score of the five-item depression and five-item anxiety subscales because they were highly correlated (average *r* = 0.69, ranging from 0.53 to 0.89; am *R*_c_ = 0.75; pm *R*_c_ = 0.70; all timepoints *R*_c_ = 0.79).

### Analytic Strategy

#### Multi-Level Modeling

Because of the multilevel structure of the data in which days were nested within couples, we conducted multilevel analyses in R (Sarkar, 2008; Wickham, 2009, 2017; Bates et al., 2015; Bates and Maechler, 2017; Kuznetsova et al., 2017; R Core Team, 2017; Revelle, 2017; Wickham and Miller, 2017). In these analyses, within-couple variation was modeled at Level 1 and between-couple variation was modeled at Level 2. We began by calculating Intraclass Correlations (ICCs) for all study variables to examine the amount of variance attributable to stable differences between couples and variance attributable to fluctuations over time within couples (see **Table 1**). ICCs were higher than 0.18 for all variables, indicating that a multilevel approach was appropriate. We also computed within-couple Pearson correlations for study variables.

**Table 1:** Descriptive statistics of study variables.

	Patient			Partner		
		
	Grand mean	*SD*	ICC	Grand mean	*SD*	ICC
**AM observations**						
AM pain	4.10	2.14	0.69	–	–	–
Esteem/emotional	3.10	0.85	0.60	2.51	0.89	0.65
Solicitous	2.22	1.03	0.64	2.11	0.95	0.62
Negative	1.10	0.30	0.29	1.14	0.27	0.18
**PM observations**						
PM pain	3.86	2.17	0.69	–	–	–
Esteem/emotional	3.18	0.83	0.60	2.60	0.89	0.78
Solicitous	2.29	1.02	0.54	2.09	0.91	0.66
Negative	1.09	0.22	0.18	1.14	0.24	0.31


*Grand means and standard deviations were calculated across all person-days.*

#### Hypothesis Testing

For our main analyses, we ran a random intercept model examining the roles of morning support receipt and provision in predicting evening pain.^3^ Morning pain was included in all models so that we could examine effects of support on residualized change in pain from morning to evening. We included both spouses’ reports of support mobilization so that we could examine the unique effects of each partner’s perspective. Following this, we added quantity of time spent with the partner, negative affect, and positive affect to the model. All predictor variables were centered on the mean for each couple (i.e., group-mean centered) so that we could examine within-couple effects.

#### Reverse Causation

We ran a series of additional models examining whether within-patient fluctuations in pain were significantly associated with subsequent residualized changes in each type of patient support receipt and spouse support provision. In these models, evening reports of each type of support were specified as a function of within-couple centered morning levels of that type of support and within-couple centered morning pain.

## Results

### Response Rate and Descriptive Statistics

Response rates on the twice-daily interviews were excellent, with 404 of the possible 406 morning and evening interviews completed by patients and 404 of 406 interviews completed by spouses. Twenty-seven of the included patients and twenty-seven of the included spouses completed all 14 interviews. Two patients and two spouses each missed one interview. With few exceptions, there were no missing items within the completed interviews. A single item was missing for patient negative affect for one of the morning interviews. We addressed this by taking the average of the remaining nine items for that interview. Patients and spouses reported not seeing each other on 4% of the half-days. In these cases, participants did not complete the support items. Therefore, some participants had fewer than 14 interviews included in the analyses if they (1) missed an interview, or (2) did not see their spouse. The restricted maximum likelihood estimation of linear mixed-effects models applied here is robust to missingness and can account for unbalanced numbers of observations per group (Bates et al., 2015; Kuznetsova et al., 2017).

**Table 1** displays descriptive statistics for study variables. As can be seen in the Table, the grand means for emotional support receipt and provision indicate that emotional support was mobilized “somewhat” – “a lot” across person-days. The grand means for solicitous support receipt and provision indicate that solicitous support was mobilized “a little” – “somewhat.” The grand means for negative support receipt and provision were low, corresponding to the “not at all” response option. We inspected frequencies of our study variables. These analyses revealed that frequencies of negative support were low, with values >1 on only 8% of the AM patient reports, 7% of the PM patient reports, 7% of the AM spouse reports, 7% of the PM spouse reports. This is a point we return to in our discussion.

Although the focus of this study is on within-couple associations among spouse responses and patient pain, for the purpose of future meta-analyses, we report within- and between-couple correlations of study variables in **Table 2**. These correlations were calculated using the statsBy function in the *psych* package in R (Revelle, 2017), which calculates the pooled within-group correlations and the sample size weighted between-couple correlations. Within-couple associations of each type of morning support and evening pain were in the expected directions. Mornings when patients received higher levels of esteem/emotional support and lower levels of solicitous and negative support than typical for them were non-significantly associated with lower levels of evening pain (for esteem/emotional support: *r* = -0.10, *p* = 0.176; for solicitous support: *r* = 0.06, *p* = 0.427; for negative support: *r* = 0.12, *p* = 0.108. Additionally, mornings when partners provided higher levels of esteem/emotional support than typical for them were non-significantly associated with lower evening patient pain, *r* = -0.07, *p* = 0.230. There were significant associations between partner reports of solicitous and negative support provision and evening patient pain: mornings when partners reported providing higher levels of solicitous and negative support than typical for them were associated with higher evening levels of patient pain (for solicitous support: *r* = 0.16, *p* = 0.030; for negative support: *r* = 0.17, *p* = 0.017). Readers should interpret the between-couple correlations with caution given the low sample size; however, the overall pattern suggests that patients who experienced higher levels of pain tended to receive higher levels of all types of support as reported by patients and partners, although most of these correlations were not significant.

**Table 2:** Within- and between-couple bivariate correlations among study variables.

	1	2	3	4	5	6	7	8	9	10	11	12	13	14
**AM patient report**														
1. Pain	-	0.05	0.20	0.13	0.07	0.42^*^	0.08	0.89^***^	0.09	0.23	0.04	0.06	0.34^+^	0.24
2. Esteem/emotional	-0.00	-	0.67^***^	-0.50^**^	0.59^***^	0.41^*^	-0.50^**^	0.16	0.85^***^	0.63^***^	-0.40^*^	0.62^***^	0.43^*^	-0.52^**^
3. Solicitous	0.07	0.30^***^	-	-0.30	0.35^+^	0.43^*^	-0.30	0.24	0.59^***^	0.91^***^	-0.17	0.38^*^	0.41^*^	-0.26
4. Negative	-0.06	-0.13^+^	0.11	-	-0.48^**^	-0.36^+^	0.70^***^	0.12	-0.49^**^	-0.36^+^	0.78^***^	-0.52^**^	-0.33^+^	0.82^***^
**AM partner report**														
5. Esteem/emotional	0.08	0.01	0.06	0.06	-	0.66^***^	-0.21	0.04	0.54^**^	0.31^+^	-0.40^*^	0.92^***^	0.67^***^	-0.47^*^
6. Solicitous	0.11	0.02	0.08	0.03	0.35^***^	-	-0.12	0.39^*^	0.33^+^	0.36^+^	-0.28	0.68^***^	0.95^***^	-0.22
7. Negative	0.12^+^	0.05	0.01	0.02	0.05	-0.03	-	-0.03	-0.44^*^	-0.38^*^	0.49^**^	-0.32^+^	-0.16	0.57^**^
**PM patient report**														
8. Pain	0.50^***^	-0.10	0.06	0.12	-0.09	0.16^*^	0.17^*^	-	0.16	0.26	0.07	0.00	0.30	0.26
9. Esteem/emotional	0.05	0.32^***^	0.11	-0.04	0.07	-0.04	-0.06	-0.06	-	0.66^***^	-0.30	0.54^**^	0.27	-0.48^**^
10. Solicitous	0.22^*^	0.15^*^	0.23^**^	-0.02	-0.00	0.00	0.02	0.17^*^	0.16^*^	-	-0.18	0.31^+^	0.23	-0.26
11. Negative	-0.15^*^	-0.13^+^	-0.12	-0.19^**^	-0.05	0.05	-0.02	-0.11	-0.12^+^	-0.09	-	-0.44^*^	-0.20	0.51^**^
**PM partner report**														
12. Esteem/emotional	0.10	0.03	-0.03	0.01	0.23^**^	0.13^+^	-0.01	-0.07	0.07	0.05	0.04	-	0.74^***^	-0.44
13. Solicitous	0.22^*^	-0.03	0.10	0.01	0.01	0.26^***^	-0.15^+^	0.22^**^	-0.04	0.22^**^	0.02	0.27^***^	-	-0.24
14. Negative	-0.04	0.04	0.06	0.06	0.01	0.01	-0.06	-0.05	0.05	0.05	0.00	0.03	0.04	-


*Pooled within-couple correlations are presented below the diagonal and sample size weighted between-couple correlations are presented above the diagonal. ^+^*p* < 0.100, ^∗^*p* < 0.050, ^∗∗^*p* < 0.010, ^∗∗∗^*p* < 0.001.*

### Cross-Day Changes in Pain

We conducted multilevel regression analysis predicting residualized change in patient pain as a function of each type of support mobilization to patients as reported by patients and partners. These results are displayed in **Table 3**. Contrary to expectations, patient reports of esteem and solicitous support were not significantly associated with subsequent changes in pain (esteem: *b* = -0.26, *SE* = 0.16, *t*(146) = 1.63, *p* = 0.105; solicitous: *b* = 0.06, *SE* = 0.14, *t*(147) = 0.45, *p* = 0.656. However, the effects of patient negative support receipt were consistent with expectations: mornings when patients reported receiving higher levels of negative support than typical for them were associated with subsequent increases in pain, *b* = 0.63, *SE* = 0.31, *t*(146) = 2.00, *p* = 0.045. Additionally, mornings when partners reported providing higher levels of esteem support and lower levels of solicitous support than typical for them were associated with subsequent decreases in patients’ pain from morning to evening (esteem: *b* = -0.48, *SE* = 0.16, *t*(146) = -2.93, *p* = 0.004; solicitous: *b* = 0.35, *SE* = 0.15, *t*(147) = 2.38, *p* = 0.019). Increases in partner negative support provision were not significantly associated with subsequent residualized increases in patient pain, *b* = 0.74, *SE* = 0.38, *t*(147) = 1.94, *p* = 0.055. When we included time spent with the partner, negative affect, and positive affect in the model, results were unchanged. We examined statistical assumptions of linearity, homoscedasticity, and normal distribution of residuals and all three assumptions were met for both models presented in **Table 3**.

**Table 3:** Predicting residualized change in patient pain from morning to evening as a function of morning esteem/emotional, solicitous, and negative support mobilized to patients as reported by patients and partners.

	Model 1			Model 2		
		
Fixed effects	Estimate (SE)	*t* (df)	*p*	Estimate (SE)	*t* (df)	*p*
Intercept	3.89 (0.36)	10.72 (28)	<0.001	3.89 (0.37)	10.69 (28)	<0.001
AM pain	0.50 (0.07)	7.56 (147)	<0.001	0.50 (0.07)	7.03 (143)	<0.001
AM time spent with spouse				-0.03 (0.13)	-0.22 (144)	0.828
AM negative affect				-0.04 (0.35)	-0.12 (143)	0.905
AM positive affect				-0.03 (0.19)	-0.16 (143)	0.874
**Patient report of support receipt**						
AM esteem/emotional	-0.26 (0.16)	-1.63 (146)	0.105	-0.26 (0.17)	-1.52 (142)	0.130
AM solicitous	0.06 (0.14)	0.45 (147)	0.656	0.05 (0.14)	0.36 (143)	0.718
AM negative	0.63 (0.31)	2.00 (146)	0.048	0.64 (0.32)	1.99 (142)	0.048
**Partner report of support provision**						
AM esteem/emotional	-0.48 (0.16)	-2.93 (146)	0.004	-0.46 (0.17)	-2.68 (142)	0.008
AM solicitous	0.35 (0.15)	2.38 (146)	0.019	0.33 (0.15)	2.22 (142)	0.028
AM negative	0.74 (0.38)	1.94 (147)	0.055	0.56 (0.42)	1.34 (143)	0.184
**Random effects**	**Standard deviation**			**Standard deviation**		
Intercept	1.907			1.908		
Residual	1.002			1.009		


*Model based on 181–182 days from 29 couples. All predictors have been centered relative to person means that were calculated based on all available observations.*

### Reverse Causation

Next, we examined whether morning-to-morning within-patient fluctuations in pain were associated with subsequent changes in each type of support in six multilevel regression models – one for each type of support (i.e., patient received esteem support, patient received solicitous support, patient received negative support, spouse provided esteem support, spouse provided solicitous support, and spouse provided negative support). Fluctuations in morning pain were not significantly associated with subsequent changes in levels of esteem support receipt reported by patients, *b* = 0.02, *SE* = 0.03, *t*(156) = 0.71, *p* = 0.482, or with subsequent changes in levels of esteem support provision reported by partners, *b* = 0.03, *SE* = 0.03, *t*(155) = 1.18, *p* = 0.240. However, increases in morning pain were significantly associated with subsequent increases in solicitous support receipt reported by patients, *b* = 0.13, *SE* = 0.04, *t*(157) = 2.94, *p* = 0.004 as well as with subsequent increases in solicitous support provision reported by partners, *b* = 0.09, *SE* = 0.03, *t*(156) = 2.68, *p* = 0.008. Finally, within-patient increases in pain were not significantly associated with decreases in patient negative support receipt, *b* = -0.02, *SE* = 0.01, *t*(158) = -1.84, *p* = 0.067. Changes in pain were not significantly associated with changes in negative support provision reported by partners, *b* = 0.001, *SE* = 0.01, *t*(158) = -0.12, *p* = 0.902.

## Discussion

We addressed the question of how spouses might best support patients coping with chronic pain. This is one of a handful of intensive longitudinal studies to address this question and this is the first to examine effects of multiple types of spouse responses on subsequent changes in pain. We found that esteem/emotional support provision by spouses was associated with subsequent decreases in pain across the day. In contrast, solicitous support provision by spouses and negative support receipt by patients were associated with subsequent increases in pain across the day. This study provides evidence that examining only total support may mask important differences in the effects of specific types. In the past studies of social support, even when differentiating types of support, have tended to lump solicitous and esteem support together, treating both as emotional support. However, consistent with operant models of pain, our study suggests that the effects of these two types of support on patient pain outcomes may be quite different. This study also provides evidence that assessing both partners’ perceptions of partner responses provides complementary information that would be missed if only one partners’ perceptions were assessed.

Our primary goal was to examine a model in which spouse responses are expected to lead to changes in patient adjustment. However, a competing model is that patient pain is independent of social influences, and that associations that have been observed in previous cross-sectional studies between patient pain and spouse responses are simply due to spouses reacting to patient disability. Previously, Burns et al. (2013) examined time-ordered associations between patient pain and spouse criticism and hostility. Specifically, they found that higher spouse hostility was associated with subsequent increases in patient pain, and patient pain was associated with subsequent decreases in spouse criticism and hostility. Similar to Burns et al’s. (2013) study, we found some evidence for bidirectional causality between negative support receipt and pain. Higher levels of negative support receipt were associated with subsequent increases in patient pain. Although patient pain was not significantly associated with subsequent change in negative support receipt with alpha set at 0.05, a more liberal alpha of 0.10 would lead to the conclusion that higher levels of patient pain were associated with subsequent decreases in negative support receipt. Future research utilizing larger samples should further examine the bidirectional association between patient pain and negative support receipt.

Our study replicates and extends the work of Burns et al. (2013), by going beyond negative spouse responses and examining esteem/emotional and solicitous spouse responses. Although we found evidence that esteem/emotional support provision by spouses may lead to decreases in patient pain, we did not find evidence that patient pain leads to shifts in esteem/emotional support mobilization to patients as reported by either the patient or the spouse. These results extend previous research indicating a general beneficial effect of esteem/emotional support to individuals with chronic illness (Weinberger et al., 1990; Rosen et al., 2014, 2015; Beggs et al., 2015; Song et al., 2015; Hemphill et al., 2016). However, this is the first study of which we are aware to examine daily time-ordered associations between esteem/emotional support and patient pain.

We found that within-couple increases in spouse reports of solicitous support provision were associated with subsequent increases in patient pain. In reverse causation analyses, we found that higher levels of patient pain were associated with subsequent increases in solicitous spouse responses as reported by both partners. These results suggest a vicious cycle of patient pain and spouse solicitousness: not only may solicitous support lead to increases in patient pain, but also increases in patient pain may lead spouses to be more solicitous.

Together, these findings point to a potential target for interventions for couples coping with chronic pain. One way that spouses could become better support providers might be to learn to change the way they respond to the patient, especially when the patient is in pain. Spouses could be taught to express love, admiration, acceptance, and confidence in the patient instead of expressing concern or worry. Changing the way that spouses react to patient pain might pave the way for better pain management. The potential vicious cycle of solicitous support and pain should be examined in future research because of potential applicability for interventions in couples coping with chronic pain.

### Future Directions

In this study, most of the patients were female and most of the spouses were male. Previous studies have found gender differences in the extent to which individuals benefit from support (Neff and Karney, 2005). Although our larger proportion of female patients and male spouses reflects the distribution of RA in the population (Public Health Agency of Canada [PHAC], 2010), future studies might aim for more equal ratios of males and females in patient and spouse roles. Aiming to recruit more equal gender ratios would improve generalizability of findings to other chronic conditions and would help to disentangle the effect of patient-partner roles from husband-wife roles.

This study focused on the within-couple relations between partner responses and pain using a small sample of RA patients and their partners. We caution readers that although this study provides initial evidence on the role of support in patient outcomes, more work is needed with larger samples to replicate these results. As is often the case in samples of non-distressed couples, there were relatively few instances of negative support. This low frequency of may have limited our power to detect some of the effects of spouse negative responses. Because of the small sample and consequently limited power for between subject analyses, we were not able to test more complex models, such as models examining aggregated averages of spouse responses on average change in patient pain from morning to evening, the unique effects of morning and evening reports of spouse responses on patient pain, or interactive effects among different spouse response variables. Future research could examine these more complex and potentially more informative models. Additionally, it was not possible to examine stable factors that might influence the extent to which partner responses were associated with changes in pain. For example, patients who are more satisfied with their relationship partner in general may not be as impacted by negative support receipt compared to those who are less satisfied with their relationship partner (DeLongis et al., 2010). Additionally, there may be differences between patients in support effectiveness depending on how much pain patients tend to experience. Future research with larger samples could examine more complex models, including stable factors that might moderate associations among the variables examined here. Importantly, however, our results were unchanged when controlling for the quantity of time spent with the spouse, negative affect, and positive affect.

Future research is needed to examine *how* spouse responses influence well-being. Our findings here suggest that solicitous and negative spouse responses lead to increases in patient pain, and esteem/emotional spouse support leads to decreases in patient pain. We propose two potential mediators to examine in future research. The first is self-esteem. Associations have previously been found between receiving support and reduced self-esteem in recipients (Nadler et al., 1983; Nadler, 1987). Fisher et al. (1982) theorized that support includes self-threatening and supportive components. They argued that the self-threatening components lead to increased psychological distress whereas the supportive components lead to decreased psychological distress. More recently, Leary (2012) theorized that self-esteem changes as a function of the extent to which people perceive that they are relationally valued and accepted by others. We propose that whether support negatively impacts self-esteem depends on the type of support being mobilized (Pow and DeLongis, 2018). Solicitous and negative support may have detrimental effects on patient well-being across studies because these forms of support both communicate that the spouse believes that the patient is struggling and may not be able to handle things on his or her own. In contrast, esteem/emotional support communicates that the patient is loved and valued and would be expected to lead to improvements in self-esteem.

Along with self-esteem, spouse responses may also influence patient well-being by altering patient perceptions of spouse responsivity, which is the perception of understanding and validation from the spouse (Reis and Shaver, 1988). Perceived spouse responsivity has been found to fluctuate across days (Laurenceau et al., 2005) and has been associated with long-term improvements in well-being (Selcuk et al., 2015; Slatcher et al., 2015). In one cross-sectional study of couples in which one partner was experiencing a lupus flare-up, spouse reports of emotional support were significantly associated with higher levels of patient perceived spouse responsiveness, which was, in turn, associated with lower patient depressive symptoms (Fekete et al., 2007). Negative support had the opposite effect. Lower levels of patient perceived spouse responsiveness mediated the positive association between spouse negative support provision and patient depressive symptoms. Although this study identified perceived spouse responsiveness as a promising potential mechanism linking spouse responses and well-being for those coping with chronic illness, research is needed that examines time-ordered associations. Intensive longitudinal studies would allow for the examination of whether spouse responses are associated with subsequent shifts in perceived spouse responsiveness, and whether these shifts in spouse responsiveness account for changes in patient pain and other indicators of well-being.

## Conclusion

Researchers typically examine coping from an individualistic perspective without examining the social context. Our findings suggest that spouse responses play a key role in promoting adaptation in individuals coping with chronic pain. Within the limitations of the current study and sample, our findings advocate for the expression of love and acceptance to individuals with chronic pain. They also advocate against expressions from the provider of worry about or criticism of the recipient.

## Ethics Statement

This study was carried out in accordance with the recommendations of The University of British Columbia’s Behavioural Research Ethics Board. The protocol was approved by The University of British Columbia’s Behavioural Research Ethics Board. All subjects gave verbal informed consent in accordance with the Tri-Council Policy Statement (T2, 2014), the International Conference on Harmonization Good Clinical Practice Guidelines (ICH-GCP) and the requirements of the U.S. Department of Health and Human Services, as set out in the Federal Policy for the Protection of Human Subjects, 45 CFR Part 46, sub-part A.

## Author Contributions

AD designed and directed the project, developed the theoretical framework, and supervised all work. JP and ES conducted the data analyses and drafted the manuscript. MH contributed to the writing of the manuscript. All authors discussed the results and contributed to the final manuscript.

## Conflict of Interest Statement

The authors declare that the research was conducted in the absence of any commercial or financial relationships that could be construed as a potential conflict of interest.

## Footnotes

1 Here we report the *R*_c_, which is the internal consistency reliability of change within persons throughout the study (Cranford et al., 2006). However, it is important to note that the items we used to assess support can be considered formative rather than reflective indicators (Bollen and Lennox, 1991). That is, reflective indicators are indicators that are “caused by” an underlying latent variable and should be highly correlated (Bollen and Lennox, 1991). However, formative indicators are conceptualized as components that “cause” or “determine” the construct and are not necessarily expected to be highly correlated (Bollen and Lennox, 1991). For example, even though complaining about one’s spouse and avoiding one’s spouse are two components that are theorized to function together as negative support (Bodenmann, 2005), they may not occur at the same time and may not be expected to be highly correlated. Previous studies have conceptualized supportive behaviors using this measurement model (Collins and Feeney, 2000).

2 An examination of the items making up spouse negative support provision indicated that the items were not correlated at the within-person level (*r* = -0.01, *ns*). Because complaining about one’s spouse and avoiding one’s spouse are two components that are theorized to function together as negative support (Bodenmann, 2005), we created a variable summing across those items despite low within-person internal consistency of the items. However, we ran an additional model to supplement our main analyses that included both items as separate within-person predictors of changes in patient pain. Neither item was significantly associated with changes in pain from morning to evening (*p*s > 0.100). Additionally, having these items in the model as separate predictors rather than as one composite predictor did not change associations we observed for the other support variables.

3 We also examined whether evening reports of support were associated with subsequent residualized change in patient pain from evening to the next morning. However, there were no significant associations of within-couple evening support receipt or provision on residualized change in well-being from evening to the next morning.
